# The Mediating Role of Anger and Anxiety in the Association of Social Support with Mobility Among Middle-Age and Older Adults with Knee Osteoarthritis

**DOI:** 10.3390/ijerph22020283

**Published:** 2025-02-14

**Authors:** Erin R. Harrell, Patricia A. Parmelee, Dylan M. Smith

**Affiliations:** 1 Department of Psychology, The University of Alabama, Tuscaloosa, AL 35401, USA; paparmelee@gmail.com; 2 Department of Family, Population and Preventive Medicine, Stony Brook University, Stony Brook, NY 11794, USA; dylan.smith@stonybrookmedicine.edu

**Keywords:** osteoarthritis (OA), anger, anxiety, mobility limitation, social support

## Abstract

Introduction: Osteoarthritis (OA) is one of the leading causes of chronic disability in older adults, often causing significant impairment of mobility. OA symptoms have been linked to mental functioning, including depression, anxiety, and negative affect. Method: To examine whether anger and anxiety mediate the relationship between social support and mobility among older adults with knee osteoarthritis, data from the Everyday Quality of Life in Older Blacks and Whites with Osteoarthritis (EQUAL) study (*N* = 336) were analyzed using Hayes’ PROCESS model in SPSS to test the direct effect of social support on mobility as well as mediation by anxiety and anger. Results: While univariate models for both anxiety and anger were significant, only anxiety mediated the relationship between social support and mobility. Conclusion: Although limited by their cross-sectional nature, the findings suggest that at least part of the association of social support with mobility may be explained by the role of support in alleviating anxiety.

## 1. Introduction

Osteoarthritis (OA), the most common form of arthritis, is a leading cause of chronic pain, mobility limitations, and long-term disability [1,2]. By 2050, it is estimated that there will be 642 million individuals with knee osteoarthritis, 279 million with hand osteoarthritis, and 62.6 million with hip osteoarthritis, with another 118 million individuals presenting with other types of osteoarthritis [3]. As the most common form of osteoarthritis, knee osteoarthritis has known risk factors including female sex, high physical demanding occupational load, joint injury, overweight and obesity, and older age [4,5]. Given the forecasted increase in our aging population combined with an increased proportion of individuals in the general population who are obese or overweight, the prevalence of knee osteoarthritis is only expected to rise [3,6]. Knee osteoarthritis has not only been linked to weight gain but also joint trauma due to repetitive movements (e.g., squatting, kneeling) resulting in profound effects on quality of life among both younger and older adults alike. Recently, there has been an increase in the number of younger adults affected by osteoarthritis, with the fastest growth in total knee replacement rates being among persons less than 60 years of age [7].

Studies have shown that those living with knee osteoarthritis may experience decreased movement and mobility [8], resulting in poorer physical and mental functioning [9]. Knee osteoarthritis also has a social impact [10,11,12] accounting for more disability among older adults than any other disease [13,14]. Fortunately, for over 20 years, researchers have consistently reported that social support is beneficial for health and may act as an appropriate buffer against psychological distress induced by chronic health conditions like knee osteoarthritis [15]. Social support, provided in an adequate format and to an appropriate degree such that it addresses the chronic pain sufferer’s needs, has been shown to have both direct and buffering effects on psychological well-being [16]. When an individual is experiencing decreased mobility, as is typical with knee osteoarthritis, having someone provide tangible or emotional support may buffer some of the negative consequences of major illness or stressful situations [17]. For example, Rivera and colleagues [18] examined the relationships of social interactions with daily pain and mood symptoms in people with osteoarthritis of the knee and found that social interactions have the potential not only to influence the mood of adults with OA of the knee but to attenuate the negative impact that chronic pain has on mood symptoms.

To explore the relationship between emotional distress in individuals with knee osteoarthritis and the social support available to them, we take a biopsychosocial approach. The biopsychosocial model of knee osteoarthritis emphasizes both psychologic and social factors and their impact beyond the biological presentation of individuals with knee OA to include its effects on mobility and daily activities. Specifically, psychologic factors such as depression and anxiety [19], anxiety and pain catastrophizing [20], and fear avoidance behaviors linked to anxiety are associated with more severe pain and decreased engagement in social activities that require one to be more mobile [21]. In addition, individuals with limited social support systems may potentially result in a pathway model in which a decline in social support, in relation to what is needed to compensate for functional limitations, such as mobility challenges or disability, will trigger feelings of anxiety and anger, which, in turn, exacerbate mobility limitations. Given the link between mobility and the ability to be independent, this is consistent with findings that have linked the inability to be independent with feelings of anxiety, anger, and helplessness [22].

### Emotional Affect, Chronic Pain, and Social Support

The experience of chronic pain may also provoke underlying tendencies towards depression, which has been linked to feelings of helplessness, poor coping ability, and sleep disturbances [23,24]. This link has led to the development of hypothetical pathway models that illustrate the vicious cycle that osteoarthritis can create by linking knee osteoarthritis to pain and depression/anxiety to excess disease burden. Research in this area has shown that social support via emotional or tangible assistance can mediate the relationship between pain and depressive symptoms [25,26].

While psychological correlates of depression and anxiety are highly implicated in the pain disability cycle [27], anger has received less attention. An integral part of the pain experience, anger is a powerful predictor of prevailing and future symptoms and outcomes in patients with knee osteoarthritis [28,29]. Higher anger expression is not only commonplace among chronic pain sufferers but is also associated with poorer functional outcomes such as physical performance deficits like mobility limitations [30]. In addition, reduced social support has been linked to both aggressive anger expression (anger-out) and anger suppression (anger-in) in a variety of populations with health problems [31,32,33,34,35].

Feelings of anger can arise in patients with knee OA because chronic pain patients often perceive themselves as victims of injustice, which, in turn, may foster disturbed therapeutic relations with treatment providers as well as individuals in their social environment [36]. These negative outcomes may also serve to increase emotions such as anxiety due to anxiety being the more common emotion and anger being a response that some people exhibit when they experience anxiety [22].

Prior work has demonstrated an association of unmet social needs with a higher incidence of mental health symptoms like anxiety and decreased levels of capability [37]. Research has shown that anxiety can heighten osteoarthritis pain and disability while lowering quality of life [37,38]. Therefore, adequate social support to help meet social needs, like job and food insecurity and transportation needs, may, in turn, serve to lessen anxiety and help one cope with mobility limitations [38]. This study employs secondary data analyses to test whether the impact of social support on mobility is mediated by emotional well-being (anger and anxiety). It is hypothesized that social support will be associated with greater mobility, with this relationship being mediated by reduced feelings of anger and anxiety.

## 2. Materials and Methods

The mediation analysis for this study used data from the “Everyday Quality of Life in Older Blacks and Whites with Osteoarthritis (EQUAL) Study”, a multi-site study of adults 40 years and older with a clinical diagnosis of knee osteoarthritis [18,39]. The EQUAL Study was approved by the Institutional Review Boards at Stony Brook University and The University of Alabama. Informed consent was obtained during the initial interview, and documentation is on file.

### 2.1. Sample and Recruitment

Participants were recruited from two sites, western Alabama and Long Island, New York, beginning in 2013 with follow-up completed in 2019. Since participants had to have a clinical diagnosis of osteoarthritis, most participants were recruited from outpatient and geriatric specialty clinics. At both sites, medical chart reviews to identify eligible patients were supplemented by flyers/posters placed in clinic waiting and exam rooms. For specialty clinics, the research team stationed research assistants (RAs) in waiting rooms during peak hours to work with staff to identify potential participants. All prospective participants then received a letter briefly describing the study, either mailed under the signature of the attending physician or hand delivered during a regular clinic visit. Exclusion criteria were (1) significant cognitive impairment, evidenced by four or more errors (10-point scale) on the Short Portable Mental Status Questionnaire (SPMSQ; [40]; (2) life-threatening illness (e.g., cancer other than basal cell, heart or lung disease that requires home use of oxygen); (3) diagnosed rheumatoid arthritis, fibromyalgia, or other rheumatologic disease, to avoid confounding with OA symptoms; and (4) sensory or language problems that preclude completion of interviews in English.

Once participants were consented, The EQUAL Study team conducted personal interviews with the sampled population in addition to administering written questionnaires. Given the focus on the association of social support and mobility, only data from participants who completed both the social provisions scale and life-space assessment measure, described below, were used in the analysis, resulting in a sample size of *N* = 336.

### 2.2. Measures and Procedures

#### 2.2.1. Background Characteristics

Background characteristics were self-reported and included as potential covariates. These were race (Black vs. non-Hispanic White; other racial groups were excluded per aims of the larger study), age, sex, and education (6-point scale, “less than high school” through “graduate degree”). Physical health was represented by a count of chronic and acute conditions experienced over the past year on a 28-item checklist (e.g., heart trouble, diabetes, broken hip) including certain chronic diseases that have been suggested to affect osteoarthritis (e.g., obesity, hypertension, and diabetes mellitus). Pain was assessed with the pain subscale of the Knee injury and Osteoarthritis Outcomes Score (KOOS) [41]. Cronbach’s alpha for the pain scale was 0.89.

#### 2.2.2. The Life-Space Assessment (LSA)

The Life-Space Assessment (LSA), developed by Baker and colleagues [42], was used to assess mobility, which is a crucial component to independent living. The LSA derives a score based on reported geographic movement during the four weeks prior to the assessment. The measure comprises five life-space levels: (1) other rooms of your home besides the room where you sleep; (2) an area outside your home such as your porch, deck or patio, hallway (of an apartment building), or garage in your own yard or driveway; (3) places in your neighborhood, other than your own yard or apartment building; (4) places outside your neighborhood, but within your town; and (5) places outside your town. Participants were asked to indicate how many times they visited a location: less than once a week; 1–3 times each week; 4–6 times each week; or daily. The assessment also assesses whether they required assistance from another individual or an assistive device. Once completed, LSA scores can range from 0 (indicating that a person is totally room bound) to 120 (the person travelled out of town every day without assistance). Thus, the lower the score, the more diminished the physical mobility and the greater the mobility limitations of the individual.

#### 2.2.3. Social Support

The Social Provisions Scale [43] is a 24-item measure comprising six subscales, each containing four items. The subscales are guidance, reassurance of worth, social integration, attachment, and nurturance and reliable alliance. All items are measured on a 4-point scale and ask the participant to rate the extent to which they feel each statement describes their current relationship with other people. If a person feels a statement is very true of their current relationships, they will respond with a 4 (strongly agree). If they feel that the statement does not clearly describe their relationship, they will respond with a 1 (strongly disagree). Thus, a high score indicates that the individual is receiving more support. For the current analysis, we used the scale total, computed by averaging across items with reverse coding as necessary, allowing for 20% missing items. Cronbach’s alpha for this sample was 0.923.

#### 2.2.4. Emotional Well-Being

State Anxiety was measured using the six-item short form of the Spielberger State Trait Anxiety Inventory developed by Marteau and Bekker [44]. This shortened version produces scores similar to that of the full form [45] for subjects experiencing normal and raised levels of anxiety. Each item has four response categories (“not at all”, “somewhat”, “moderately”, and “very much”), which are assigned numerical values (1–4). Higher scores reflect greater anxiety. State Anger was measured using the Trait Anger Scale [46] and comprised a 10-item Likert-type scale (1 = almost never to 4 = almost always) on which participants reported how angry they generally felt. Cronbach’s alphas for the anxiety inventory and anger scale were 0.84 and 0.89, respectively.

### 2.3. Statistical Approach

**Figure 1:**
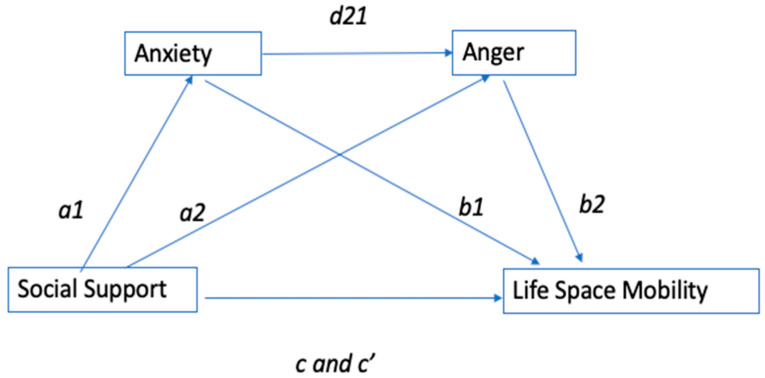
Serial multiple mediation model with paths.

Statistical analysis was conducted using version 27 of the IBM SPSS Statistics for Windows. We did not conduct a power analysis for the models reported here, as they were not part of the original proposal. However, the sample size *N* = 336 falls within the range recommended for medium-sized effects in mediational analysis [47]. After descriptive statistics were conducted for all measures, Pearson correlations were run to examine raw associations among study variables. Next, a series of linear regressions was performed to determine covariates for the main analyses. For these preliminary analyses, covariates were added to the model using a blockwise methodology in which sociodemographic variables (age, sex, race, and education) were added first, followed by health measures (health conditions and OA pain). These variables were included as covariates if they significantly and independently predicted the dependent variable, life-space mobility. After determining significant covariates, primary analyses were conducted using Hayes’ SPSS PROCESS Version 4.0 [48]. To identify significant covariates, baseline demographic characteristics (age, sex, race, and highest education level) were entered into the first step of a blockwise multiple regression analysis with LSA scores as the outcome. OA pain and general health status were then entered at step 2. Model 6 within Hayes Process was then used to test a serial multiple mediation as diagrammed in Figure 1 with social support as the independent or predictor variable, anxiety as the first mediator, anger as the second mediator, and life-space mobility as the dependent or outcome variable. Model 6 allows for testing of a serial mediation model where the mediators are ordered between the predictor and the outcome variable. In our serial mediation model, anxiety preceded anger in the model due to anxiety being the more common emotion with anger being a response that some people exhibit when they experience anxiety [22]. The pathways in the model are also labeled to reflect hypothesized causal reference between the mediators, predictor variable, and outcome variable such that a1 represents the path between social support and anxiety, a2 represents the path between social support and anger, b1 represents the path between anxiety and life-space mobility, b2 represents the path between anger and life-space mobility, c and c′ represent the path between social support and life-space mobility, and d21 represents the path between anxiety and anger (see Figure 1).

## 3. Results

### 3.1. Participants

Participants’ ages ranged from 48 to 97 years, with the average age being 64 years. Of the 336 participants who completed the entire study, 77% identified as female; 44.9% (*N* = 151) identified as Black, and 55.1% (*N* = 185) identified as White. Approximately 45% of participants reported being coupled or partnered with another person. Only 10% of the sample indicated that they did not complete high school or obtain a GED. Two participants did not provide information regarding education level.

### 3.2. Descriptive Analyses

Descriptive statistics appear in Table 1 followed by raw correlations among study variables of interest in Table 2.

### 3.3. Covariate Analysis

A significant step 1 equation (adjusted R^2^ = 0.21, Δ R^2^ = 0.22, F[4, 329] = 23.36, *p* < 0.001) was attributable to level of education (b = 0.23, SE = 0.97, *p* < 0.001), sex (b = −0.13, SE = 3.76, *p* = 0.007), age (b = −0.16, SE = 0.18, *p* = 0.001), and race (b = −0.28, SE = 3.29, *p* < 0.001). The addition of OA pain and general health status at step 2 significantly improved prediction (adjusted R^2^ = 0.42, Δ R^2^ = 0.21, F[2, 327] = 60.79, *p* < 0.001), reflecting a positive association of pain (b = −0.34, SE = 1.62, *p* < 0.001) and general health status (b = −0.28, SE = 0.58, *p* < 0.001) with life-space mobility.

### 3.4. Serial Mediation Analyses

Anxiety and anger were examined as serial mediators of the relationship between social support and functional mobility within one’s life space or environment. All paths of the serial mediation models are illustrated in Figure 1. The model was first examined without covariates. The overall models were significant for both emotions: anxiety, F(1, 334) = 29.51, *p* < 0.001, R^2^ = 0.08; and anger, F(2, 333) = 119.3, *p* < 0.001, R^2^ = 0.42. The hypothesized indirect effect of anxiety and anger on mobility was not significant (a1d21b2 = 0.29, SE = 0.90, 95% CI [−1.59, 2.00]). However, the specific indirect effect of anxiety was significant (a1b1 = 3.24, SE = 1.42, 95% CI [0.86, 6.40]), showing that anxiety mediates the relationship between social support and mobility. The specific indirect effect of anger only (a2b2), however, was not significant, showing that anger did not mediate the relationship between social support and mobility.

Next, serial mediation was repeated with the covariates. When accounting for these covariates, the overall model was significant for the mediating effect of anxiety F(7, 326) = 18.32, *p* < 0.001, R^2^ = 0.28. In this model, we see that social support predicts anxiety b = −0.36, t(326) = −5.33, *p* < 0.001 (a1); see Table 3.

For the proximal mediator of anger with covariates included, the overall model is also significant F(8, 325) = 31.74, *p* < 0.001, R^2^ = 0.44. However, the only significant predictor in this model is anxiety b = 0.41, t(325) = 12.06, *p* < 0.001 (d21); see Table 3. While there were not serial indirect effects of anxiety and anger (a1d21b2), the specific direct effect of anxiety remained significant, a1b1 = 2.40, SE = 1.11, 95% CI [0.45, 4.84].

As anticipated, the direct effect (c′) of social support on life-space mobility was positive and statistically significant (b = 9.97, t(324) = 3.75, *p* = 0.002). The direct effect (b1) of anxiety on life-space mobility was negative and significant (b = −6.75, t(324) = −2.65, *p* = 0.0084), indicating that anxiety predicts life-space mobility. The direct effect (b2) of anger on life-space mobility was positive but not significant (b = 0.80, t(324) = 0.23, *p* = 0.819), indicating that anger does not predict life-space mobility; see Table 3.

In summary, significant covariates were age, race, health conditions, and OA pain. All of these covariates bore negative relations to mobility, indicating lower mobility among persons who were older, self-identified as Black/African American, and had more health problems and OA pain. Regarding the net of these effects, the relationship of social support with mobility reflected a significant direct effect. Parsing of the significant indirect effect using bootstrapping methods revealed that the overall significant effect was driven by the path from social support, through anxiety, directly to mobility. Table 4 shows the total, direct, and indirect effects of social support on mobility as well as the covariates used in the serial mediation.

## 4. Discussion

Our analysis indicates that both social support and negative emotions are associated with mobility limitations, defined here in terms of the distance in the environment that an individual is able to navigate outside of one’s central hub. Given that the positive influence of a social network can provide motivation and, possibly, means to resume community mobility [49], having limited social support was hypothesized to impact anxiety, which has similar characteristics to stress [50] including irritability, fatigue, and muscle pain [50]. The results of the current study support prior research linking social support to anxiety and mobility limitations where the receipt of pain-related emotional support has been linked to better psychological well-being [51].

Not only did our model show that social support predicts anxiety, but it also demonstrated that the relationship between social support and mobility limitation is mediated by anxiety. In addition, the hypothesis that anxiety and anger would serve as serial mediators, such that individuals with greater anxiety would also have greater anger impacting the relationship between social support and life-space mobility, was partially supported. The mediation models for both anxiety and anger as respective outcome variables were both significant when control variables were not included in the model. This was to be expected given that anxiety and anger were highly correlated. However, once we added control variables, only the indirect effect of anxiety remained significant. This suggests that individuals with knee osteoarthritis who are experiencing chronic pain may also benefit from other social support, including psychotherapy to help them develop healthy coping mechanisms for dealing with their anxiety and other negative emotions, like anger and irritability. This is especially important for individuals with comorbidities, as negative emotions have been thought to lead to damage to one’s physical and mental health by elevating cardiovascular reactivity (Broaden and Build Theory; [52]).

### Limitations and Future Directions

Some limitations of this study should be noted. First, anxiety and anger were assessed via self-reports, which could have been biased given that both are typically associated with stigmas that people high on these traits are physically aggressive or in need of anger-management training [53,54]. In addition, the measures for anxiety and anger did not specifically question participants about their anxiety/anger over their OA. Similarly, although our measure of social support captures a broad range of supportive functions others may serve, it does not depict the range of interpersonal dynamics that may be relevant to coping with chronic pain. In particular, the Social Provisions Scale focuses solely on positive supportive assistance provided by others; neither negative social interactions nor support provided to others are captured. Both are central to the dynamic linkage of support, negative emotions, and mobility limitations; this is a promising area for future research. It should be noted that, because the data are cross-sectional, these analyses cannot speak to potential causal mechanisms underlying observed associations among social support, negative emotional well-being, and mobility limitations. Nor is our proposed mediational model, with anger and (more strongly, anxiety) mediating the support–mobility association, the only possible interpretation of the linkages among these variables. Future research should also consider investigating anxiety and anger as moderators. Acknowledging that further longitudinal work is needed to clarify the exact dynamics at work here, it would also be helpful for future investigators to examine how changes in support over time affect the mobility and emotional well-being of older adults with osteoarthritis-related functional limitations.

## 5. Conclusions

Although limited by their cross-sectional nature, the findings suggest that at least part of the association of social support with mobility may be explained by the role of support in alleviating anxiety. If clinicians have a better understanding of the impact of psychosocial factors and how they influence patient outcomes in individuals diagnosed with knee osteoarthritis, this may help with being able to provide better screenings of patients in clinical settings and provide more individualized recommendations for coping. Having a greater awareness of the lived experiences of individuals living with knee osteoarthritis can also lead to better treatment outcomes as well as identifying areas of social support that can be most effective for the individual.

## Figures and Tables

**Table 1:** Demographic characteristics of baseline sample.

	Black/African American			White		
	Alabama	New York	Total	Alabama	New York	Total
	*N* = 97	*N* = 54	*N* = 151	*N* = 88	*N* = 98	*N* = 186
	*N* (%)	*N* (%)	*N* (%)	*N* (%)	*N* (%)	*N* (%)
Age M (SD)	63.2 (8.7)	62.7 (7.6)	63.0 (8.3)	66.0 (10.2)	63.0 (8.0)	64.4 (9.2)
Male ^R1^	14 (14.4)	12 (22.2)	26 (17.2)	24 (27.3)	26 (26.5)	50 (26.9)
Female	83 (85.6)	42 (77.8)	125 (82.8)	64 (72.7)	72 (73.5)	136 (73.1)
11 years or fewer ^R3, L1^	20 (20.8)	5 (9.4)	25 (16.8)	7 (8.0)	2 (2.0)	9 (4.8)
High school/GED	29 (30.2)	12 (22.6)	41 (27.5)	16 (18.2)	18 (18.4)	34 (18.3)
Vocational/technical	13 (13.5)	6 (11.3)	19 (12.8)	8 (9.1)	9 (9.2)	17 (9.1)
Some college	17 (17.7)	12 (22.6)	29 (19.5)	12 (13.6)	21 (21.4)	33 (17.7)
College degree	7 (7.3)	9 (17.0)	16 (10.7)	18 (20.5)	24 (24.5)	42 (22.6)
Graduate degree	10 (10.4)	9 (17.0)	19 (12.8)	27 (30.7)	24 (24.5)	51 (27.4)

R = race L = location 1 = *p* < 0.05 2 = *p* < 0.01 3 = *p* < 0.001.

**Table 2:** Correlation matrix of key variables.

		Life-Space Total	Anger Mean	Anxiety Mean	Social Provisions Scale Mean	Sex	Age	Highest Level Education	Race	OA Pain Mean	Health Conditions Score
Life-Space Total		1	−0.234 **	−0.307 **	0.385 **	−0.204 **	−0.140 *	0.325 **	−0.341 **	−0.499 **	−0.498 **
	*N*	337	337	337	336	337	337	335	337	337	337
Anger Mean		−0.234 **	1	0.643 **	−0.246 **	0.068	−0.194 **	−0.178 **	0.002	0.291 **	0.230 **
	*N*	337	337	337	336	337	337	335	337	337	337
Anxiety Mean		−0.307 **	0.643 **	1	−0.285 **	0.165 **	−0.171 **	−0.127 *	−0.096	0.363 **	0.236 **
	*N*	337	337	337	336	337	337	335	337	337	337
Social Provisions Scale Mean		0.385 **	−0.246 **	−0.285 **	1	−0.049	−0.098	0.319 **	−0.173 **	−0.159 **	−0.351 **
	*N*	336	336	336	336	336	336	334	336	336	336
Sex		−0.204 **	0.068	0.165 **	−0.049	1	0.035	−0.115 *	0.115 *	0.196 **	0.176 **
	*N*	337	337	337	336	337	337	335	337	337	337
Age		−0.140 *	−0.194 **	−0.171 **	−0.098	0.035	1	0.004	−0.080	−0.078	0.115 *
	*N*	337	337	337	336	337	337	335	337	337	337
Highest Level Education		0.325 **	−0.178 **	−0.127*	0.319 **	−0.115 *	0.004	1	−0.292 **	−0.332 **	−0.346 **
	*N*	335	335	335	334	335	335	335	335	335	335
Race		−0.341 **	0.002	−0.096	−0.173 **	0.115 *	−0.080	−0.292 **	1	0.175 **	0.202 **
	*N*	337	337	337	336	337	337	335	337	337	337
OA Pain Mean		−0.499 **	0.291 **	0.363 **	−0.159**	0.196 **	−0.078	−0.332 **	0.175 **	1	0.390 **
	*N*	337	337	337	336	337	337	335	337	337	337
Health Conditions Score		−0.498 **	0.230 **	0.236 **	−0.351 **	0.176 **	0.115 *	−0.346 **	0.202 **	0.390 **	1
	*N*	337	337	337	336	337	337	335	337	337	337

Note: * *p* < 0.05. ** *p* < 0.01. We examined the distributions of all of our continuous variables, and while some (e.g., anger), showed a positive skew, this did not appear to substantively affect the findings (e.g., Spearman correlations produced the same significant associations as in Table 2, and were all of similar magnitude).

**Table 3:** Path coefficients from serial mediation models including anger.

	Anxiety		
Path	b	SE	*p*
	Without covariates (*N* = 336)		
a1	−0.36	0.28	<0.001 **
a2	−0.06	0.04	0.12
d21	0.43	0.03	<0.001 **
b1	−8.89	2.94	0.003 **
b2	−1.83	4.24	0.67
c′	18.48	2.97	<0.001 **
	With covariates (*N* = 334)		
a1	−0.36	0.07	<0.001 **
a2	−0.04	0.04	0.41
d21	0.41	0.03	<0.001 **
b1	−6.75	2.55	0.008 **
b2	0.80	3.48	0.82
c′	9.97	2.67	0.002 **

Note: *N* = 334 with covariates added due to two participants not entering in information for education level. Added covariates were age, sex, race, education level, health conditions, and osteoarthritis pain. Note: * *p* < 0.05. ** *p* < 0.01.

**Table 4:** Covariates used in serial mediation: total, direct, and indirect effects of social support on mobility.

Variable	b	SE	t	*p*	Lower CI	Upper CI
Age	−0.465	0.151	−3.08	0.003		
Sex	−4.516	3.190	−1.42	0.158		
Race	−13.922	2.761	−5.04	0.001		
Education	−0.120	0.879	−0.14	0.892		
Health conditions	−2.820	0.587	−4.81	0.001		
OA pain	−11.603	1.572	−7.38	0.001		
Social Support						
Total effect	13.035	2.632	4.95	0.001		
Direct effect	10.765	2.717	3.96	0.001		
Indirect effect	2.271	0.937			0.595	4.317
Indirect Path 1	2.424	1.149			0.424	4.931
Indirect Path 2	−0.030	0.256			−0.677	0.475
Indirect Path 3	−0.123	0.642			−0.151	1.050
Indirect Path 1: a1 **→** b1 Indirect Path 2: a2 **→** b2 Indirect Path 3: a1 **→** d21 **→** b2						

Note: Results of the final serial mediation model, as depicted in Figure 1, are summarized in Table 3. Indirect path 1: a1 > b1 = social support > anxiety > life-space mobility. Indirect path 2: a2 > b2 = social support > anger* > life-space mobility. Indirect path 3: a1 > d21 > b2 = social support > anxiety > anger* > life-space mobility. * Anger did not play a significant mediating role either alone or as a serial mediator.

## Data Availability

If requested, the data utilized in the current study are available from Dr. Dylan Smith (email: dylan.smith@stonybrookmedicine.edu) and will be provided on a case-by-case basis. The specific hypotheses for this study were not preregistered.
